# Genetic variation of the bronze locus (*MC1R*) in turkeys from Southern Brazil

**DOI:** 10.1590/1678-4685-GMB-2016-0136

**Published:** 2017-03-20

**Authors:** Josmael Corso, Diego Hepp, Mônica C. Ledur, Jane O. Peixoto, Nelson J. R. Fagundes, Thales R. O. Freitas

**Affiliations:** 1 Departmento de Genética, Universidade Federal do Rio Grande do Sul (UFRGS), Porto Alegre, RS, Brazil; 2 Instituto Federal de Educação, Ciência e Tecnologia do Rio Grande do Sul (IFRS), Porto Alegre, RS, Brazil; 3 Embrapa Suínos e Aves, Concórdia, SC, Brazil

**Keywords:** Meleagris gallopavo, white phenotype, melanocortin-1 receptor, bronze locus, plumage color

## Abstract

Domestic turkeys present several color phenotypes controlled by at least five genetic loci, but only one of these has been identified precisely: the bronze locus, which turned out to be the *melanocortin-1 receptor* (*MC1R*) gene. *MC1R* variation is important for breeders interested in maintaining or developing different color varieties. In this study, we sequenced most of the *MC1R* gene from 16 White Holland (the main commercial turkey variety) and 19 pigmented turkeys from southern Brazil with two purposes. The first was to describe the *MC1R* diversity in White Holland turkeys, which may serve as reservoirs of genetic diversity at this locus. The second was to test whether the traditional color classification used by Brazilian breeders is related to previously known *MC1R* alleles. White Holland turkeys had four different haplotypes corresponding to the bronze (*b* ^+^) and black-winged bronze (*b* ^1^) alleles. Pigmented turkeys also had four haplotypes corresponding to the *b* ^*+*^ and *b* ^1^ alleles, but different haplotypes represent the most common *b* ^*+*^ allele in these two groups. The black (*B*) allele was absent from our samples. Overall, our results suggest that white and pigmented individuals form two different populations, and that the traditional color classification used by Brazilian breeders cannot accurately predict the genotypes at the bronze locus.

The turkey (*Meleagris gallopavo*) is a bird with high commercial value, playing a preponderant role in the Brazilian poultry industry, once Brazil is the second largest producer and exporter of turkey meat (Antunes, 2005; Lima, 2014). Domestic turkeys have been bred into many varieties with different plumage patterns, and have been used in classical genetic studies to determine the genetic basis of color phenotypes (Robertson *et al.*, 1943). At least five loci were characterized as responsible for the great color variation in this species (Robertson *et al.*, 1943). One of these loci, the bronze locus, shows three alleles in dominance order: *B* (black) > *b* ^+^ (wild bronze) > *b* ^1^ (black-winged bronze) (Asmundson, 1945). Recently, Vidal *et al.* (2010) showed that the bronze locus is actually the melanocortin-1 receptor gene (*MC1R*), with the Black and black-winged bronze phenotypes being considered as a case of dominant or recessive melanism, respectively. *MC1R* is known as a key regulator of melanin synthesis and has been associated to melanism in several vertebrate species (Mundy, 2005; Skoglund and Höglund, 2010).

Molecular characterization of the *MC1R* gene can be useful in breeding programs aiming at maintaining or developing different color varieties in species such as turkeys, in which the different and exquisite plumages have ornamental value. Turkeys bred in backyards for ornamental or family consumption purposes usually have predominantly dark plumage, while turkeys raised for the poultry industry have white plumage, with White Holland being one of the major commercial varieties used in Brazil. White turkeys have the recessive allele *c* in homozygosity in an epistatic locus for color, and therefore may have any allele combination in the bronze locus (Robertson *et al.*, 1943; Asmundson, 1945). Even though white turkeys may serve as a reservoir of *MC1R* variation to be used in turkey breeding programs, DNA sequence diversity of this locus has never been studied in commercial varieties. Likewise, turkey breeders from Southern Brazil use an informal, though traditional, classification of turkey color patterns based on general plumage features, and, therefore, it is unknown whether this classification is useful for predicting genotypes or alleles present in the bronze locus. In this study, we performed the first characterization of the *MC1R* locus in White Holland turkeys from Southern Brazil. In addition, we characterized the *MC1R* variation found in colored turkeys raised in backyards in Southern Brazil to test the predictive potential of the informal phenotypic classification made by breeders concerning the bronze locus. Understanding the genetic variation at the bonze locus may assist small producers in developing breeding strategies focused in producing feathers with decorative commercial value.

We analyzed 35 domestic turkeys, being 16 White Holland and 19 colored birds (Table 1). We used two alternative phenotypic classifications. First, we used the informal traditional classification attributed by breeders in Southern Brazil. Second, we asked Mr. Kevin Porter (Porter's Rare Heritage Turkeys breeding facility, IN, USA) to classify these birds in standard varieties based on pictures taken during sampling collection. DNA was extracted from blood placed on paper filter with the extraction method modified from Boyce *et al.* (1989). The sampling procedure involved a small punction in the foot of the birds, and was carried out after informed consent given by the birds’ owners. About 2-4 adult birds were sampled in different properties, which were separated by ~2-15 km among each other. Colored turkeys were sampled in small farms around the city of Concórdia, in Santa Catarina state, while White Holland turkeys were sampled in small farms around the city of Antonio Prado, in the Rio Grande do Sul state, Brazil. These two locations are separated by approximately 200 km. All experimental protocols employed in the present study that relate to animal experimentation were performed in accordance with the protocol number 010/2012 approved by the Embrapa Swine and Poultry Ethics Committee on Animal Utilization, following national and international guidelines for animal welfare.

**Table 1:** Molecular characterization of the turkey *melanocortin-1 receptor* gene (*MC1R*) and phenotype classification using the breeders’ traditional color classification and Porter's Rare Heritage standard classification.

	Substitution^A^							*MC1R* haplotype^B^	Bronze locus allele	Color Phenotype^C^	
	90	**96**	186	**364**	411	450	**887**		
	G30	W32*	L62	**I122F**	A137	Y150	**A296V**	
Sample ID	C	**G**	C	**A**	C	C	**C**	H1 (MC1R*1)	*b* ^+^		
·	**·**	·	**T**	·	T	**·**	H2 (MC1R*2)	*B*	Tradicional	Porter's	
·	**A**	·	**T**	·	T	**·**	H3 (MC1R*3)	*b1*	Color	Standard	
·	**·**	·	**·**	·	T	**T**	H4 (MC1R*4)	*b1*	Classification	Classification	
·	**·**	·	**·**	·	T	**·**	H5 (MC1R*5)	*b* ^+^			
	·	**·**	G	**·**	·	·	**·**	H6	*b* ^+^		
	?	**?**	G	**·**	T	·	**·**	H7	*b* ^+^		
	A	**·**	·	**·**	·	·	**·**	H8	*b* ^+^		
Per01	·	**·**	C/G	**·**	·	·	**·**	H1/H6	*b* ^+^ *b* ^+^	White	–
Per02	?	**?**	G	**·**	T	·	**·**	H7/H7	*b* ^+^ *b* ^+^	White	–
Per03	·	**·**	C/G	**·**	·	·	**·**	H1/H6	*b* ^+^ *b* ^+^	White	–
Per04	·	**·**	C/G	**·**	C/T	·	**·**	H1/H7	*b* ^+^ *b* ^+^	White	–
Per06	·	**·**	C/G	**·**	C/T	·	**·**	H1/H7	*b* ^+^ *b* ^+^	White	–
Per07	?	**?**	C/G	**·**	·	·	**·**	H1/H6	*b* ^+^ *b* ^+^	White	–
Per10	·	**·**	·	**·**	·	·	**·**	H1/H1	*b* ^+^ *b* ^+^	White	–
Per11	·	**·**	C/G	**·**	·	·	**·**	H1/H6	*b* ^+^ *b* ^+^	White	–
Per12	C/A	**·**	·	**·**	·	·	**·**	H1/H8	*b* ^+^ *b* ^+^	White	–
Per13	·	**·**	C/G	**·**	C/T	·	**·**	H1/H7	*b* ^+^ *b* ^+^	White	–
Per14	·	**·**	·	**·**	·	·	**·**	H1/H1	*b* ^+^ *b* ^+^	White	–
Per17	·	**·**	·	**·**	·	·	**·**	H1/H1	*b* ^+^ *b* ^+^	White	–
Per21	·	**·**	C/G	**·**	C/T	·	**·**	H1/H7	*b* ^+^ *b* ^+^	White	–
Per22	·	**·**	·	**·**	·	·	**·**	H1/H1	*b* ^+^ *b* ^+^	White	–
Per24	?	**·**	·	**·**	·	C/T	**C/T**	H1/H4	*b* ^+^ *b1*	White	–
Per25	?	**?**	C/G	**·**	·	·	**·**	H1/H6	*b* ^+^ *b* ^+^	White	–
Pp02	?	**?**	·	**·**	·	T	**T**	H4/H4	*b1b1*	*Carijó*	Tricolor
Pp03	?	**?**	·	**·**	·	T	**C/T**	H4/H5	*b* ^+^ *b1*	*Preto*	Bronze
Pp04	?	**?**	·	**·**	·	T	**T**	H4/H4	*b1b1*	*Carijó*	Tricolor
Pp06	?	**?**	G	**·**	·	·	**·**	H6/H6	*b* ^+^ *b* ^+^	*Preto*	Bronze
Pp09	?	**?**	·	**·**	·	T	**T**	H4/H4	*b1b1*	*Carijó Marrom*	Tricolor
Pp10	?	**?**	C/G	**·**	C/T	C/T	**C/T**	H4/H7	*b* ^+^ *b1*	*Carijó Marrom*	Bronze
Pp11	·	**·**	·	**·**	·	T	**T**	H4/H4	*b1b1*	*Carijó*	Tricolor
Pp13	·	**·**	C/G	**·**	·	C/T	**C/T**	H4/H6	*b* ^+^ *b1*	*Carijó*	Bronze
Pp14	?	**?**	·	**·**	·	T	**T**	H4/H4	*b1b1*	*Carijó Marrom*	Tricolor
Pp15	?	**?**	·	**·**	·	T	**C/T**	H4/H5	*b* ^+^ *b1*	*Carijó*	Red Bronze
Pp16	?	**?**	C/G	**·**	·	C/T	**C/T**	H4/H6	*b* ^+^ *b1*	*Carijó*	Red Bronze
Pp17	?	**?**	·	**·**	·	T	**C/T**	H4/H5	*b* ^+^ *b1*	*Carijó*	Bronze
Pp18	?	**?**	·	**·**	·	T	**·**	H5/H5	*b* ^+^ *b* ^+^	*Preto com Vermelho*	Bronze
Pp22	·	**·**	·	**·**	·	T	**T**	H4/H4	*b1b1*	*Carijó*	Tricolor
Pp24	·	**·**	·	**·**	·	T	**T**	H4/H4	*b1b1*	*Carijó*	Tiger Bronze
Pp28	?	**?**	·	**·**	·	T	**T**	H4/H4	*b1b1*	*Carijó*	Royal Palm
Pp30	?	**?**	·	**·**	·	T	**·**	H5/H5	*b* ^+^ *b* ^+^	*Preto*	Bronze
Pp31	?	**?**	·	**·**	·	T	**T**	H4/H4	*b1b1*	*Carijó Marrom*	Tricolor
Pp34	?	**?**	C/G	**·**	C/T	C/T	**C/T**	H4/H7	*b* ^+^ *b1*	*Carijó*	Red Bronze

A *MC1R* nucleotide position is shown above, while the protein position and the corresponding amino acid substitution is shown below. Missense and nonsense mutations are shown in bold. Note that the protein coded by MC1R*3 is truncated at 31 amino acids due to a premature stop codon. Identical nucleotides are represented by “.”. Missing data are represented by “?”.

B Haplotypes H1-H5 according to Vidal *et al.* (2010), Haplotypes H6-H8 are new haplotypes identified in this study. For individuals having missing data, haplotypes consider imputed genotypes.

C White Holland birds have not been subjected to further phenotypic classification based on photographs.

A fragment of the *MC1R* gene was amplified with primers MC1R5UTRF1MG 5’-CTG GCT GAG GCC GGG GCC-3’ and MC1R3UTRR1MG 5’-GAG GTC GGG CAG CCC AAC-3’ (Vidal *et al.*, 2010). PCR reactions contained 2-4 μL of DNA template, 0.25 mM of each dNTP, 1 x PCR Buffer, 1.5 mM MgCl_2_, 0.1 units of *Taq* DNA polymerase, 0.25 μm of each primer and ddH2O to a final volume of 25 μL. After an initial denaturation step at 94 °C for 5 min, we performed 35 cycles at 94 °C for 30 s, 61.5 °C for 40 s and 72 °C for 30 s, followed by a final extension step at 72 °C for 5 min. PCR products were checked in agarose gels (1%), purified with Exonuclease I and Shrimp Alkaline Phosphatase (GE Healthcare), and sequenced by Macrogen (Seoul, South Korea) with the primers used during PCR amplification. Sequences with 831 to 1036 bp of the *MC1R* coding region were deposited in GenBank under accession numbers KP867098-KP867115. Nucleotide sequences were aligned and translated using MEGA 5 (Tamura *et al.*, 2011). Putative heterozygous sites were identified by visual inspection of the chromatograms and confirmed by sequencing the complementary strand. Haplotypes for heterozygous individuals were resolved using the PHASE algorithm implemented in the software program DNAsp v5 (Librado and Rozas, 2009). PHASE implements a Bayesian statistical method for reconstructing haplotypes from population genotype data (Stephens *et al.*, 2001). In short, a Markov chain Monte Carlo (MCMC) sampler was used to explore alternative haplotype combinations to find the most likely candidates under the implemented population genetics model and return their posterior distribution conditioned on genotype data. The MCMC sampler was run for 100,000 iterations, sampling every 100 iterations. The first 10,000 iterations were discarded as burn-in.

Five variable sites were detected, representing four synonymous and one nonsynonymous substitution (Table 1). When compared to *MC1R* haplotypes previously characterized in this species (Vidal *et al.*, 2010), our study found three known (H1, H4, and H5) plus three new haplotypes (H6-H8), which differ from known haplotypes by synonymous substitutions. Thus, all new haplotypes represent variants of the wild-type bronze allele *b* ^+^. Despite the small sample size, haplotype estimation was conservative concerning new haplotypes. Haplotypes H6 and H7 were found in homozygous birds, and H8 was found in a single heterozygous, in which haplotypes are unambiguous. We could not determine positions 90 and 96 for all individuals. As a result, haplotype pairs H1xH8 and H2xH3 would be indistinguishable in these cases. Indeed, three white birds (Per7, Per24 and Per25) identified as heterozygous for haplotype H1 (plus H4 or H6) could be heterozygous for H8 (plus H4 or H6). However, because H1 and H8 are alternative versions of the *b* ^+^ allele, this uncertainty is not relevant for our study. On the other hand, for colored birds all haplotypes found in our sample were independent of these two positions, leading to unambiguous haplotype estimates. Table 2 shows the haplotype frequency for white and colored turkeys based on PHASE. Among white individuals, we found a single *b* ^1^ allele, with all other alleles being *b* ^+^ variants. On the other hand, in colored individuals, *b* ^1^ had a frequency of ~66%, with the remaining alleles being *b* ^+^ variants. We did not find any *B* allele in our samples. Curiously, the most frequent haplotype for a *b* ^+^ allele in white birds (H1) has not been found in colored birds. In addition, the most frequent haplotype in colored turkeys (H4) was almost absent in White Holland (Table 2).

**Table 2:** *MC1R* haplotype frequencies in sampled individuals. Absolute numbers are given in parenthesis.

Haplotype	Bronze locus allele	White turkeys	Colored turkeys
H1	*b* ^+^	0.594 (19)	0.000 (0)
H4	*b* ^1^	0.031 (1)	0.658 (25)
H5	*b* ^+^	0.000 (0)	0.184 (7)
H6	*b* ^+^	0.156 (5)	0.105 (4)
H7	*b* ^+^	0.188 (6)	0.053 (2)
H8	*b* ^+^	0.031 (1)	0.000 (0)

Color phenotypes based on the traditional color classification included *Carijó* (which corresponds to a Mottled/Flecked color pattern), *Carijó-Marrom* (Mottled/Flecked Brown), *Preto com Vermelho* (Black with Red) and *Preto* (Black) (Supplementary material Figure S1). This classification scheme had a poor performance for predicting genotypes at the bronze locus. For example, *Carijó* and *Carijó Marrom* included birds having either *b* ^+^ *b* ^1^ or *b* ^*1*^ *b* ^1^ genotypes. Birds classified as *Preto com Vermelho* or *Preto*, for which a *B* allele would be expected, were all *b* ^+^ *b* ^+^. On the other hand, a standard phenotypic classification performed by Mr. Kevin Porter matched perfectly the expected and observed *MC1R* genotypes. Only phenotypes expected to be *b* ^+^ *b* ^+^ or *b* ^+^ *b* ^1^ (Bronze and Red Bronze), or *b* ^*1*^ *b* ^1^ (Royal Palm, Tricolor, and Tiger Bronze) were identified among the birds included in this study (Table 1).

Genetic diversity analyses were performed using software Arlequin 3.5 (Excoffier and Lischer, 2010) considering the whole *MC1R* gene fragment as a “standard data”, in which each haplotype represents a different allele, and all alleles are equivalent in terms of molecular distance. Therefore, all statistics reflect genetic variation at *MC1R* as a whole, rather than being an average over (linked) segregating sites. These options also minimize the problems of missing data in some sequences and of haplotype history as a confounding factor in haplotype distribution among populations.

Our analyses revealed higher observed (H_O_) and expected heterozygosity (H_E_) in white turkeys (H_O_ = 0.688, H_E_ = 0.605) compared to colored birds (H_O_ = 0.368, H_E_ = 0.533). In spite of the limited sample size within each group, these estimates resulted in a negative though non-significant inbreeding coefficient for White Holland birds (F_IS_=-0.142, p = 0.894), but in a positive and significant one for birds raised in backyards (F_IS_ = 0.315, p = 0.049). One possibility to account for these results is that while commercial birds have been bred using different “stocks” to avoid inbreeding depression (Costa F., 2006, PhD Thesis, Universidade Federal Fluminense, Niterói, RJ, Brazil), backyard turkeys tend to breed among themselves in small flocks. A non-excluding alternative is that the different properties raising colored birds are genetically structured, resulting in a heterozygote deficit due to the Wahlund effect (Wahlund, 1928). Another hypothesis is that the existence of selection driven by color in the backyard turkeys could increase the homozygosity in *MC1R*. This latter hypothesis is, however, unlikely to be true because there is no relationship between *MC1R* variation and the color phenotypes ascertained by breeders. Nonetheless, the reasons for this may be more complicated. First, because *MC1R* could be involved in color polymorphism through epistatic interaction with other loci (Hepp *et al.*, 2016), and second, because MC1R may have pleiotropic effects over other phenotypes of interest (see Ducrest *et al.*, 2008). Hence, characterizing additional genetic markers will be necessary to discriminate among these alternatives. The significant genetic structure between the commercial and “backyard” stocks (F_ST_ = 0.404, p < 0.001) corroborates the genetic distinctiveness between them, as indicated by haplotype frequency (Table 1).

In conclusion, our study found new variants of the wild-type allele at the bronze *locus* in turkeys coming from both commercial and non-commercial properties in Southern Brazil. We also showed that almost all white turkeys were homozygous for the *b* ^+^ allele. Taken together, these results suggest that White Holland turkeys are unlikely to provide an input of new bronze alleles in breeding programs aimed at producing new colored varieties. In addition, we showed that the traditional classification used by backyard turkey breeders from Southern Brazil is unrelated to the genotypes at the bronze locus, *MC1R,* which may be relevant for ornamental turkey breeding programs in this region.

## Supplementary material

The following online material is available for this article:

Figure S1 - Turkey phenotypes used in the *MC1R* characterization
