# Supplementary material for: Genetic variation of the bronze locus (MC1R) in turkeys from Southern Brazil
Source: Genet Mol Biol. 2017 Mar 20;40(1):104–8. doi: 10.1590/1678-4685-GMB-2016-0136 (PMC5409775; doi:10.1590/1678-4685-GMB-2016-0136)
Supplement: Supplementary file 1 [file 1415-4757-gmb-1678-4685-GMB-2016-0136-Suppl01.pdf]

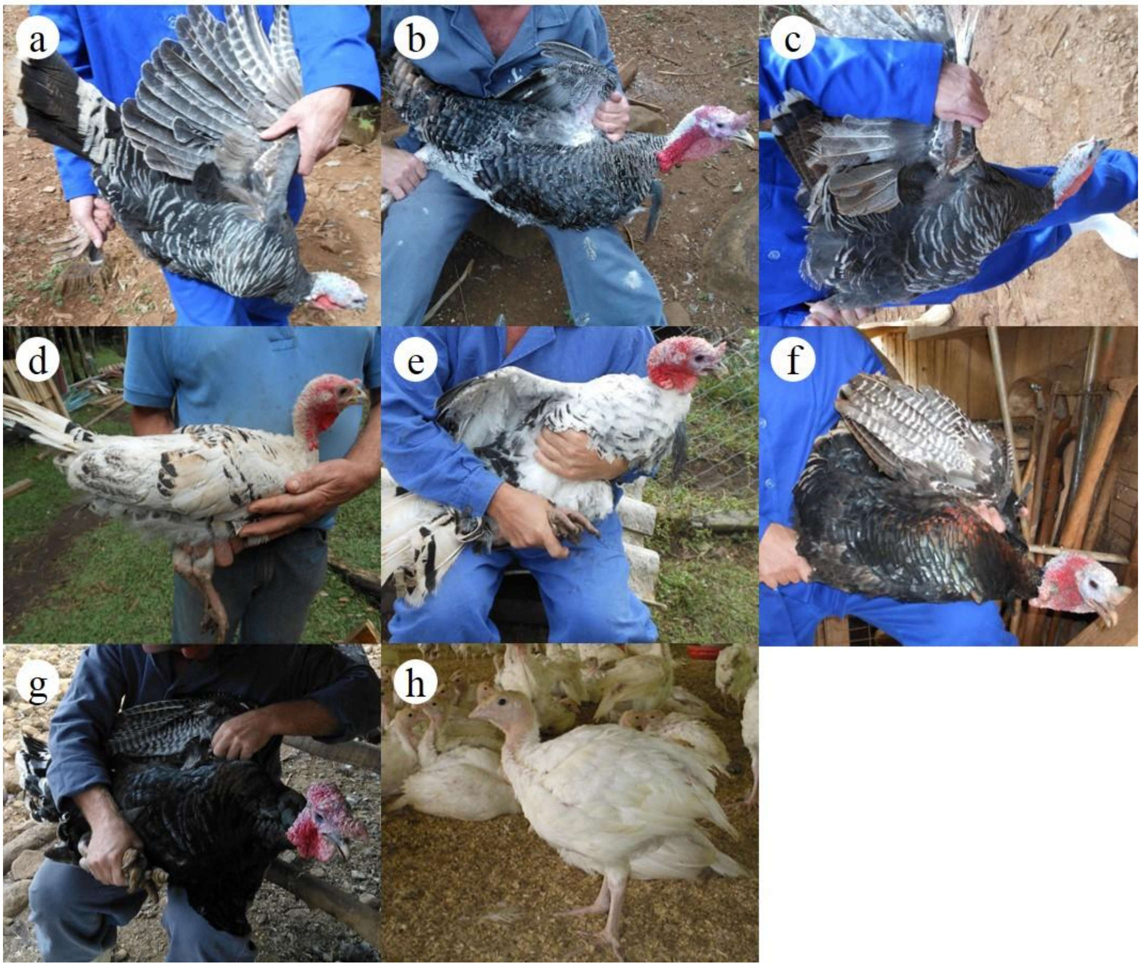

Figure S1 - Photographs from individual turkeys used in this study. Sample ID is followed by the phenotype using the traditional color classification and Porter's Rare Heritage standard classification: a) Pp17,*Carijó*/Bronze; b) Pp24, *Carijó*/Tiger Bronze; c)Pp15, *Carijó*/Red Bronze; d) Pp11, *Carijó*/Tricolor; e) Pp28, *Carijó*/Royal Palm; f) Pp18, *Preto com Vermelho*/Bronze; g) Pp06, *Preto*/Bronze; h) Per07, White/White Holland.
